# A systematic review of pharmacist input to metabolic syndrome screening, management and prevention

**DOI:** 10.1007/s11096-020-01084-3

**Published:** 2020-06-30

**Authors:** Rana Moustafa Al AdAwi, Derek Stewart, Cristin Ryan, Antonella Pia Tonna

**Affiliations:** 1 Hamad General Hospital, Hamad Medical Corporation, PO Box 6536, Doha, Qatar; 2 College of Pharmacy, QU Health, Qatar University, PO Box 2713, Doha, Qatar; 3 College of Pharmacy, Trinity College Dublin, Dublin, Ireland; 4 School of Pharmacy and Life Sciences, Robert Gordon University, Aberdeen, AB10 7AQ Scotland, UK

**Keywords:** Management, Metabolic syndrome, Pharmacist role, Prevention, Screening, Systematic review

## Abstract

*Background* Metabolic syndrome is a cluster of factors that increase the risk of cardiovascular disease and include: diabetes and prediabetes, abdominal obesity, elevated triglycerides, low high-density lipoprotein cholesterol and high blood-pressure. However, the role of the pharmacist in the metabolic syndrome has not yet been fully explored. *Aim of the review* This systematic review aimed to critically appraise, synthesise, and present the available evidence on pharmacists’ input to the screening, prevention and management of metabolic syndrome. *Method* The final protocol was based on the “Preferred Reporting Items for Systematic Review and Meta-Analysis Protocols (PRISMA-P)”. Studies published in English from January 2008 to March 2020 reporting any pharmacist activities in the screening, prevention or management of metabolic syndrome were included. Databases searched were Medline, Cumulative Index to Nursing and Allied Health Literature, International Pharmaceutical Abstracts, Cochrane and Google Scholar. Studies were assessed for quality by two researchers, data extracted and findings synthesised using a narrative approach. *Results* Of the 39,430 titles reviewed, ten studies were included (four were randomised controlled trials). Most studies focused on pharmacist input to metabolic syndrome screening and management. Screening largely involved communicating metabolic parameters to physicians. Management of metabolic syndrome described pharmacists collaborating with members of the multidisciplinary team. A positive impact was reported in all studies, including achieving metabolic syndrome parameter goals, reverting to a non-metabolic syndrome status and, improved medication adherence. The populations studied were paediatrics with risk factors, adults with comorbidities and psychiatric patients. Integration of the pharmacist within the multidisciplinary team, an easy referral process and accessibility of service were potential facilitators. Inadequate funding was the key barrier. *Conclusion* The studies describing pharmacist input in metabolic syndrome provide limited evidence of positive outcomes from screening and management as part of collaborative practice. Further work is required to provide more robust evidence of effectiveness and cost-effectiveness while considering key barriers.

**Electronic supplementary material:**

The online version of this article (10.1007/s11096-020-01084-3) contains supplementary material, which is available to authorized users.

## Impacts on Practice

- There is a lack of evidence on the role for the pharmacist in metabolic syndrome (MetS), the viable models of care and the beneficial population.
- The review suggests that pharmacists can effectively screen patients for MetS, and participate in the prevention and management amongst most at-risk populations and within different settings to enhance patient care.
- Further research is warranted to determine the readiness and acceptance for the pharmacist to intervene in MetS from the pharmacists, physicians and patient’s perspective.

## Introduction

The International Diabetes Federation (IDF) estimates that metabolic syndrome (MetS) affects one-quarter of the world’s population, doubling the risk of coronary heart disease, increasing the risk of mortality secondary to coronary heart disease by three-fold and increasing the risk of developing Type 2 diabetes mellitus (DM) fivefold [1]. MetS is a global concern due to its increasing prevalence, primarily due to obesity [1]. As a result, a number of international organisations including the IDF, the American Heart Association, the National Heart, Lung and Blood Institutes (NHLBI), the World Heart Federation, the International Atherosclerosis Society and the International Association for the Study of Obesity harmonised the criteria for metabolic syndrome (MetS). The health consequences associated with MetS are significant, [2, 3] as are the direct and indirect burdens on the healthcare system and society more generally [4–8].

It is estimated that more than 80% of cardiovascular disease (CVD) complications secondary to MetS can be prevented by optimizing blood pressure and lipid profiles [9], with the most effective, evidence-based measures being lifestyle interventions usually increased physical activity and adopting a healthier diet [10–13]. Pharmacological treatment is considered in cases of failure of non-pharmacological interventions in achieving modifiable risk factor reduction [14, 15].

To date, most evidence on health professional input to MetS management has centred on physicians and nurses [16]. While these roles are well-defined, there is less evidence supporting pharmacist involvement in MetS management. There is potential for pharmacists to apply their expert medication knowledge and clinical skills to enhance the care of patients who are at risk of or have established MetS.

The clinical and patient-facing roles of the pharmacist in the management of acute and chronic conditions have developed significantly since the seminal publication by Hepler and Strand [17]. This introduced the concept of pharmaceutical care, which was a paradigm shift from dispensing functions towards more proactive, collaborative roles in eliminating preventable drug-related problems (DRPs) [17]. Several systematic reviews and meta-analyses have provided substantial evidence of the impact of pharmacist’s intervention relating to specific MetS risk factors. Four systematic reviews and a meta-analysis of randomised controlled trials (RCTs) reported the effectiveness of the pharmacists’ interventions in the management of Type 1 and 2 DM, reducing HbA1c levels and improving medication and lifestyle adherence following pharmacist intervention [18]. Further systematic reviews and meta-analyses have provided evidence of reduction in systolic and diastolic blood pressure in hypertension (HTN) [19, 20], weight loss in obesity [21, 22] and reduction in CVD related hospitalization and mortality [23].

Furthermore, there is evidence of positive impact in other chronic conditions, including asthma and chronic obstructive pulmonary disease (COPD) [24, 25].

Growing evidence has suggested that collaborative multidisciplinary care approach in many disciplines is best practice [26]. This supports achieving the aim of better population health, better patient experience and low per capita cost [27].

A preliminary search of the Cochrane Library of Systematic Reviews and Meta-analysis and the International Database of the Prospectively Registered Systematic Review in Health and Social Science (PROSPERO) using the terms, ‘pharmacist’ and ‘metabolic syndrome’, yielded no related published or ongoing systematic reviews. A search in Medline using the same terms identified a body of primary literature sufficient for a systematic review to be undertaken.

## Aim of the review

The aim of this systematic review was to critically appraise, synthesise, and present the evidence on pharmacists’ input to the screening, prevention and management of MetS. Specific objectives were to determine the types of pharmacist input reported in the studies; determine the impact of the reported input; characterise the populations who could benefit most from the input; and identify the facilitators and barriers to the effective implementation of pharmacist input.

## Ethics approval

The Institutional Review Board (IRB) of the Medical Research Committee at Hamad Medical Corporation (HMC) in Qatar has confirmed that no ethics approval is required since this is a review.

## Method

### Protocol development

The systematic review protocol was developed based on the Preferred Reporting Items for Systematic Review and Meta-Analysis Protocols (PRISMA-P) 2015 guidance [28] (Online Appendix A) and registered in the International Prospective Register of Systematic Reviews (PROSPERO) [29].

### Inclusion and exclusion criteria

The standard systematic review PICO (population, intervention, comparator and outcomes) approach was employed [30].

#### Type of participants

All studies irrespective of population groups were included in the review.

#### Type of interventions

All pharmacist activities in the screening, prevention or management of MetS were included.

#### Type of comparator

All studies were included whether or not there was a control group comparing the impact with or without a pharmacist’s input.

#### Type of outcome

All studies were assessing the pharmacists’ input in the screening, management and prevention of MetS.

The outcomes were diverse and included the following: comparisons of different models of pharmacist input in MetS, descriptions of the process of development of the models, and the clinical outcomes of such interventions.

#### Types of studies to be included

All studies were included irrespective of design. The initial search indicated that the first relevant article was published in 2008; hence, all studies published between 2008 and March 2020, in the English language were included.

### Exclusion Criteria

Grey literature was excluded due to the potentially limited quality and difficulties in searching and retrieval [31].

### Search strategy and data sources

The electronic search strategy was guided by the “Peer Review of Electronic Search Strategies” (PRESS) checklist [32]. An initial search of Medline and Cumulative Index to Nursing and Allied Health Literature (CINAHL) was conducted, using keywords of ‘pharma*’ AND ‘metabolic syndrome’ to identify further keywords and search terms. The search string then applied to Medline, CINAHL, Cochrane, International Pharmaceutical Abstracts (IPA) and Google Scholar was (‘Metabolic syndrome*’ OR ‘syndrome x’ OR ‘Insulin resistance syndrome*’ OR ‘Dysmetabolic syndrome*’ OR “Hypertriglyceridemic waist*’ OR ‘Obesity syndrome*’ OR ‘Metabolic Cardiovascular Syndrome’ OR ‘Reaven Syndrome X’ OR ‘Atherothrombogenic syndrome’) AND ‘Pharm*’.

The reference lists of all identified articles were hand searched to identify any further relevant articles. Attempts were made to contact corresponding authors where data were missing or incomplete.

### Quality assessment and data extraction

Eligible studies were assessed for quality using standardised quality assessment tools, the Cochrane bias assessment tool and the National Heart, Lung and Blood Institute (NHLBI) quality assessment tools [33, 34]. Quality assessment using these tools was undertaken independently by two reviewers, with any disagreements resolved by discussion and referral to a third reviewer if necessary. RCTs were deemed of good quality if all criteria were of low bias risk as judged by the assessor, fair if the study had one high bias risk or two uncertain bias criteria, and poor if two or more high or uncertain bias criteria [33].

A data extraction tool was developed by adapting and customizing the “Data collection form for intervention review—RCTs and non-RCTs” from the Cochrane Collaboration [35]. Information was extracted by two independent reviewers.

### Data synthesis

Given the lack of homogeneity of study aims, participants and outcome measures, a narrative approach to data synthesis was undertaken, using text and tables aligned to each of the review objectives.

## Results

### The results of the search process

**Fig. 1:**
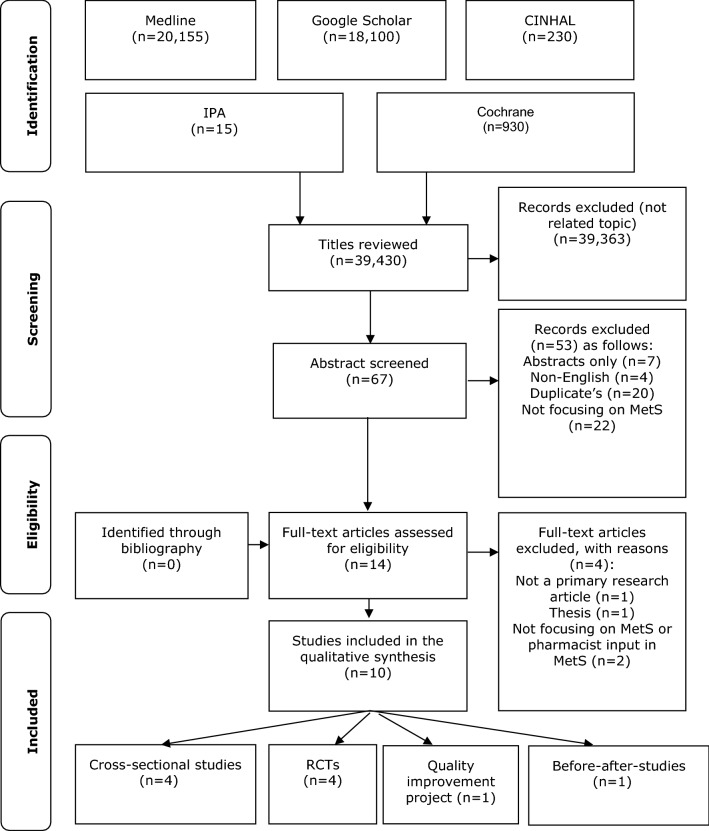
Flow diagram of the literature review process

The initial search yielded 39,430 studies. Screening of the titles excluded 39,363 titles, and abstract screening excluded a further 53. Of the 14 remaining studies, four were excluded on full-text review. Of the ten studies included in the following stages four were RCTs; four were cross-sectional design, one a before-and-after study and one was quality improvement project (Fig. 1).

### Quality assessment

Quality assessment of all included studies is reported in Tables 1 and 2. The RCTs had low bias for the primary outcome measures (Table 1). The key limitation of the cross-sectional studies was the absence of a rationale for, or calculation of, sample size (Table 2). The overall potential risk of bias of the before-and-after study was assessed to be low. Restricting the assessment to a single time point (measuring the outcome once after the outreach visits) and single hospital ward (the study was held in a psychiatric ward of the hospital) were key study limitations (Table 2).

**Table 1:** Quality assessment of randomised controlled trials studies [33]

	Hammad et al. 2011 [38]	Plaster et al. 2012 [39]	Schneiderhan et al. 2014 [40]	Azevedo et al. 2017 [44]
Random sequence generation (selection bias)	L	L	L	L
Allocation concealment (selection bias)	L	L	L	L
Blinding of participants and personnel (performance bias)	NA	NA	NA	NA
Blinding of outcome assessment (detection bias)	L	L	L	L
Incomplete outcome data (attrition bias)	L	L	L	L
Selective outcome reporting? (reporting bias)	L	L	L	L
Other bias	L	L	L	L
The quality rating	Good	Good	Good	Good

*L* low risk, *H* high risk, *NA* not applicable, *U* unclear

**Table 2:** Quality assessment of cross-sectional studies [34], and before-and-after (pre-post) studies with no control group [34] respectively

	Schneiderhan et al. 2009 [41]	Olenak and Calpin 2010 [36]	Benavides et al. 2011 [42]	Via-Sosa et al. 2014 [37]	Kjeldsen et al. 2013 [43]
*Cross*-*sectional studies*					
1. Was the research question or objective in this paper clearly stated?	Y	Y	Y	Y	
2. Was the study population clearly specified and defined?	N	Y	Y	Y	
3. Was the participation rate of eligible persons at least 50%?	CD	CD	Y	CD	
4. Were all the subjects selected or recruited from the same or similar populations (including the same time period)? Were inclusion and exclusion criteria for being in the study prespecified and applied uniformly to all participants?	Y	Y	Y	Y	
5. Was a sample size justification, power description, or variance and effect estimates provided?	N	N	N	Y	
6. Were the outcome measures (dependent variables) clearly defined, valid, reliable, and implemented consistently across all study participants?	Y	Y	Y	Y	
*Before*-*and*-*after (pre*-*post) studies*					
1. Was the study question or objective clearly stated?					Y
2. Were eligibility/selection criteria for the study population prespecified and clearly described?					Y
3. Were the participants in the study representative of those who would be eligible for the test/service/intervention in the general or clinical population of interest?					Y
4. Were all eligible participants that met the prespecified entry criteria enrolled?					CD
5. Was the sample size sufficiently large to provide confidence in the findings?					CD
6. Was the test/service/intervention clearly described and delivered consistently across the study population?					Y
7. Were the outcome measures prespecified, clearly defined, valid, reliable, and assessed consistently across all study participants?					Y
8. Were the people assessing the outcomes blinded to the participants’ exposures/interventions?					N
9. Was the loss to follow-up after baseline 20% or less? Were those lost to follow-up accounted for in the analysis?					NA
10. Did the statistical methods examine changes in outcome measures from before to after the intervention? Were statistical tests done that provided *P* values for the pre-to-post changes?					Y
11. Were outcome measures of interest taken multiple times before the intervention and multiple times after the intervention (i.e., did they use an interrupted time-series design)?					N
12. If the intervention was conducted at a group level (e.g., a whole hospital, a community, etc.) did the statistical analysis take into account the use of individual-level data to determine effects at the group level?					N

*Y* Yes, *N* No, *NA* not applicable, *CD* cannot determine

### Data extraction

Most of the studies (n = 7) were conducted in South and North America, with two in Europe and one in the Middle East. A total of 1728 participants were included, with sample size ranging from 17 to 650. The four RCTs evaluated the impact of pharmacist contribution in MetS management versus conventional care. One before-and-after study assessed the impact of a pharmacist outreach visit to implement a MetS screening program in a hospital ward. The four cross-sectional studies mainly assessed the usefulness or the implementation of pharmacist-led Mets screening. The quality improvement project evaluated the patient load before and after expansion, with special focus on the type of pharmacist input. Table 3 describes the study findings in relation to the review objectives.

**Table 3:** Description of studies included in the systematic review, in relation to the SR objectives

Author, year, country	Aim/objectives, as described by the author(s)	Study design	Results	Population	Main findings related to the reviews’ objectives			
					Description of pharmacist input	Impact of the identified input	Population who would benefit most from input	Facilitators and barriers to the effective implementation of MetS
Schneiderhan et al., 2009, Minnesota [41]	“To assess the usefulness of a metabolic risk screening program, including point-of-care (POC) glucose testing, to quantify baseline metabolic risk in outpatients receiving antipsychotics”	Retrospective cross-sectional	Of 92 patients screened 65 (71%) had one MetS parameter and 40 (43%) had 2. No significant difference reported between parameters irrespective of antipsychotic. No results presented for POC testing	Adult outpatients receiving antipsychotic medications	Piloted MetS risk screening clinic to identify rates of metabolic abnormalities Developed a MetS screening tool for patients treated with antipsychotic agents Communicated relevant information and recommendations to patients and patient’s psychiatrist	Earlier detection of Mets through pharmacist-led screening program (71% with at least 1 MetS parameter) Earlier intervention by providing proper education to the patients and relevant healthcare referral	Females receiving antipsychotics with a BMI > 30 kg/m^2^ of African American origin	*Facilitators* Being a part of and having a defined role in the multidisciplinary team Appropriate clinic set-up including facilities for referral, clinic logistics Availability of resources, such as POC devices. The latter is of particular importance when dealing with psychiatric patients *Barriers* Difficult behaviours of psychiatric patients
Olenak and Calpin., 2010, Pennsylvania [36]	“To implement a comprehensive screening for MetS that could be duplicated in other community pharmacy settings, determine the prevalence of MetS in our local community, determine the 10-year risk of developing CHD in patients with MetS, and determine the effectiveness of an education provided by the pharmacist to encourage patients to make lifestyle changes”	Prospective cross-sectional study	Baseline screening indicated that 86 (36%) had MetS. Using Framingham risk assessment, 20 (8.3%) ware at high risk of CHD (≥ 20 10-year risk of CHD) 87% with MetS self-reported lifestyle changes following education provided by the pharmacist No further details were provided	239 volunteers over 18 years of with no history of coronary heart disease (CHD)	Baseline screening for MetS and Framingham 10-year risk assessment Provision of educational lifestyle intervention at baseline for all patients Provision of screening results to the physician Assessing uptake of lifestyle modifications suggested by a non-validated questionnaire	Earlier detection of Mets through the pharmacist-led screening program Earlier intervention by providing education to the patients and relevant healthcare referral (58% discussed their results with a physician)	The prevalence of MetS was higher in older adults aged 49.9 ± 17 years and those with DM or pre-DM	*Facilitators* Community pharmacy setting placing the pharmacist in an excellent position to provide a screening service Effective communication with physicians Patient engagement and ownership of their health Availability of POC devices Effective advertising of screening service *Barriers* Financial costs of consumables
Benavides et al., 2011, Cameron [42]	“To evaluate the role of a clinical pharmacist (CP) in screening children and adolescents for components of the MetS”	Prospective, cross-sectional study	Of the 25 participants who completed the study 1 (4%) had 3 parameters of MetS, 7 (28%) had two components, and 9 (36%) had only one Treatment recommendations were made for 17 (68%) participants and communicated to the paediatrician. All were non-pharmacological interventions	High-risk children aged 10-18 years old, of Mexican–American origin in a rural ambulatory health centre	Assessing the participants for each component of MetS Educating the participants about exercise Providing the paediatrician with screening results and management recommendations	Early detection of MetS and the components in a paediatric population (68% with at least 1 MetS parameter) Enhance the early management of MetS through the provision of treatment recommendation to the physicians Increase the patient’s knowledge and awareness of MetS Prevent the progression of the disease to overt HTN and DM Referrals to physician and dietitian for further assessment	Paediatric patients with components or risk factors of MetS like obesity, first-degree family history of DM or acanthosis nigricans	*Facilitators* Previous experience in pharmacist running of a screening clinic Effective collaboration with the physicians *Barriers* Financial costs of staff and consumables Rural areas and lack of access to healthcare provision Lack of consensus on MetS definition in children and adolescents
Hammad et al., 2011, Jordan [38]	“To describe the clinical benefits of a physician-clinical pharmacist collaboration in achieving better glycemic control and better lipid and BP measurements in patients with metabolic syndrome as defined by NCEP/ATP III guidelines”	RCT non-blinded	Of the 199 participants, when comparing the control group to the intervention group, 22 (24.7%) versus 43 (39.1%), respectively (*P* = 0.032) were shifted from the MetS to none-MetS status. There was a significant reduction in TG, and BP in the intervention group compared to the control group. 308 pharmacist interventions were provided to patients and physicians	High-risk patients identified at family medicine outpatient clinics	Recruit the identified patients with suspected MetS Interview patient prior to the appointment with the physician Develop individualised care plans in collaboration with physicians Provide medication and lifestyle modification education with handouts Provide recommendations to start new treatment and laboratory monitoring Provide patient follow-up	Improve MetS status amongst the participants in the intervention group by 39.1% Improve the elements of MetS in the intervention group including TG reduced by 15 mg/dL more than in the control group (*P* = 0.029) Improve BP reduction was significantly higher in the intervention group (SBP 12.2 ± 20 mmHg and DBP 7.2 ± 12.6 mmHg (*P* = 0.049) Increase the patient’s knowledge and awareness of MetS	Patients with hypertension and high triglycerides	*Facilitators* Effective collaboration with the physicians in assessing and managing MetS Logistical issues associated with clinic site Engaging the patient and encouraging ownership and adherence Effective patient follow-up *Barriers* Lack of availability of resources including pharmacist time Lack of the formal integration of the MetS screening and management protocols within the health system
Plaster et al., 2012, Brazil [39]	“To determine the impact of a pharmaceutical care program in a sample of public outpatients with MetS”	RCT non-blinded	96 (80%) out of 120 participants had MetS. At baseline, drug-related problems (DRPs) were identified relating to efficiency, safety and necessity. At follow up, all were improved. Improvement in the intervention group was found in all the measured parameters after 6 months from baseline. This was statistically significant for almost all parameters	Diabetic patients with MetS identified at outpatient community health centres	Baseline assessment to obtain the demographical data and identify any DRP Provide pharmaceutical interventions targeting the proper medications use and lifestyle modifications Inform the physicians of the pharmacist interventions	A significant reduction in coronary heart disease (22 ± 2 to 14 ± 2%; *P* < 0.01). 83% resolution and 100% improvement of the DRPs and optimization of drug treatment Increase the medications adherence indicated indirectly by the improvement of the clinical outcomes Improve BP -13 ± 3 mmHg (*P* < 0.05) Increase weight reduction -2.6 ± 1 kg in the intervention group	Diabetic, hypertensive obese patients	*Facilitators* Effective collaboration with the physicians Being a part of the multidisciplinary team (MDT) and having a defined role Adequate financial resource Applying a pre-defined pharmaceutical framework (Dáder method) *Barriers* Limiting the community health centre pharmacist role to dispensing Lack of resource including staff and consumables
Kjeldsen et al., 2013, Denmark [43]	“To evaluate the effect of outreach visit by clinical pharmacists to support the implementation of screening of MetS at a psychiatric ward”	Before-and-after study	Improvement in utilisation of MetS screening sheets by 45% (from 34 (36%) to 91 (81%), *P* < 0.001), Better documentation of screening values 24 (26%) to 91 (81%) (*P* < 0.001) and better identification of MetS (9.3 (10%) versus 50 (45%), *P* < 0.001)	205 Patients over 18 years with schizophrenia or affective disorders, in a psychiatric ward for at least 10 days and on antipsychotics or mood-stabilizing medicines were included in the study (93 before the outreach visit and 112 after)	Auditing physician adherence to appropriate documentation Holding a weekly conference with the physicians and nurses to discuss the audit results Providing patient-specific recommendations	Successful implementation of a psychiatric hospital-based screening program indicated as follows: Increase utilization of the screening sheets by 45% Improve the quality of the screening by 55% Earlier detection of antipsychotic-induced MetS by 35% Earlier management of the identified MetS cases amongst hospitalized psychiatric patients	Adult hospitalised patients receiving antipsychotics or mood-stabilizers	*Facilitators* Availability of appropriate documentation with audit for compliance Effective collaboration with the MDT, with a defined role *Barriers* Difficult behaviours of the psychiatric patients Lack of proper communication and/or documentation with the general practitioners in the community health centres Unavailability of the IT software to facilitate documentation and communication
Via-Sosa et al., 2014, Spain [37]	“The main aim of the study was to determine the prevalence of pre-MetS, the secondary aims were to study the presence of other cardiovascular risk factors and determine patients’ cardiovascular risk”	Cross-sectional, descriptive study	Among the 650 screened participants, 124 (21.9%) had pre-MetS. Of the study population; 319 (49.1%) were hypertensive, 262 (40.3%) had abdominal obesity, 179 (27.5%) had high FBG, 131 (20.1%) had high TG and 109 (16.8%) had low HDL-C. 27% had not been previously diagnosed with dyslipidemia or hypertension	18-65 year old adults who visited 23 community pharmacies to check for MetS risk factors	Screen of participants for pre-MetS and cardiovascular risk factors including patient interviews and measurement of appropriate metabolic parameters	Earlier detection of MetS through pharmacist-led screening program (27% neve diagnosed with HTN or dyslipidemia)	Men Older adults (age > 53 years old) with BMI > 25 kg/m^2^ Sedentary lifestyle (less than 30 min regular activity 4 to 5 times per week)	*Facilitators* Community pharmacy setting placing the pharmacist in an excellent position to provide a screening service Available resource such as POC devices *Barriers* Lack of financial support including that for staff and consumables
Schneiderhan et al., 2014, Minnesota [40]	”To determine the percentage of subjects taking antipsychotic agents who meet the criteria for MetS at baseline using POC test results. Secondary objectives included the following (1) evaluate the effectiveness of the prevision by pharmacist comprehensive medication management services regarding their ability to reduce the mean difference in number of MetS risk parameters based on POC test resulted at 6 and 12 months and (2) evaluate the overall impact of psychiatric medication therapy on MetS”	RCT non-blinded	At baseline of 120 participants screened, 106 (88.3%) had dyslipidemia, 63 (52.5%) were hypertensive and 27 (22.5%) were diabetic. No significant difference in MetS parameters between groups at 6 months and 12 months. No significant difference reported between parameters irrespective of antipsychotic	Patients 18 years and over, taking antipsychotic medications recruited from three community mental health clinics who had never been reviewed by a pharmacist	Baseline assessment of MetS risk factors amongst patients receiving antipsychotic medications Assessment of the safety and effectiveness of the prescribed medications Follow up of patients at regular intervals Provision of the interpretation of POC test results, care plans, and recommendations to the physician	Earlier detection of MetS through pharmacist-led screening program allowing earlier management Potential decrease drug-induced MetS amongst psychiatric patients receive antipsychotics	Psychiatric patients who are receiving antipsychotics	*Facilitators* Availability of the POC devices particularly due to the challenging behaviour of psychiatric patients Being a part of the multidisciplinary team (MDT) with a defined role *Barriers* Challenging behaviours of psychiatric patients Lack of financial support including that for staff and consumables
Azevedo et al. 2017, Brazil [44]	“To evaluate the effectiveness of home pharmaceutical interventions in Brazilian primary care patients with MetS”	RCT non-blinded	63 patients with MetS were enrolled in the study. 64.5% (n = 49) of pharmacists’ interventions were educational and behavioural orientation. 26.3% (n = 20) involved physician requesting review, and 9.2% (n = 7) of the cases were referred to the physician for further assessment. After 6 months follow-up, the intervention group showed a significant reduction of BP by 8%, TG by 18.7%, DRPs by 59% and adherence increment by 18.2%	Adult patients aged 18 years and over diagnosed with MetS within 30 days	Baseline assessment of both groups Monthly follow-ups of the intervention group, including the following activities: Reviewing medications, identification of DRPs that might decrease the adherence and resolving them Provide education about administration and storage Diet and lifestyle recommendations Identify any unaddressed medical problem	Improve the management of MetS and the individual components (reduction of BP by 8%, TG by 18.7%) Foster medication adherence by 18.2% Decrease the DRPs by 59%	Older patients with MetS mean age 62 years particularly if (low income and low educational level)	*Facilitators* Collaboration with MDT Applying standardized care pharmaceutical care plan [61] The settings of home visits *Barriers* No identified barriers
Ganzer, Nicole 2015, West Palm Beach [45]	To evaluate the number of pharmacologic pharmacist interventions, assess the number of nonpharmacologic interventions, and compare the patient load post expansion to the pilot implementation of the metabolic clinic.	Quality improvement project	The initial pilot clinic had 40 referrals, of them 25 were followed up. The new expanded clinic received 28 referrals with 17 followed up. Twenty-five pharmacological (initiate new medications or dose adjustment) and 33 nonpharmacological interventions (diet and exercise) were made. Three referrals to national weight loss program and 1 referral to smoking secession were offered by the pharmacists	Adults diagnosed with MetS and on a second generation antipsychotic	Baseline assessment s for MetS Ordering and monitoring lab investigations Made required medications adjustments to the doses of psychiatric medications and, diabetes, hypertension and dyslipidemia medications Educating participants about diet and healthy life style, and referred to the national program for weight loss Advising patients to stop smoking and referral to smoking secession clinic	Potential improvement in management of MetS in psychiatric patients on second generation antipsychotics	Adults on a secondary generation antipsychotic with average age 58 years, male gender, smokers and not formally active	*Facilitators* Appropriate clinic set-up including facilities for referral, clinic logistics Effective advertising of screening service via in-service presentation Authority of pharmacist to initiate medications and order labs Availability of appropriate documentation Effective follow-up *Barriers* Lack of adherence to the clinic follow-up Lack of awareness of this service in the community Difficult behavior of psychiatric patients

### Data synthesis

#### Review objective 1: Description of pharmacist input

Study settings were community pharmacies [36, 37] ambulatory outpatient clinics (family medicine, community health centres and psychiatric clinics) [38–42], and pharmacist outreach visits to psychiatric patients in a hospital ward setting [43] and as part of a home healthcare service [44].

The pharmacist input was described in all the studies with varying levels of detail provided. Pharmacist screening of participants for MetS against validated criteria was described in eight studies [37–43] with screening results communicated to the relevant physician [41, 42]. In two studies, screening of other CV risk factors, using the Framingham risk assessment tool, was additionally reported [36, 37]. Various approaches to participant recruitment were described: clinic referral-based recruitment in three studies [41, 42, 45], appointment booking following appropriate advertisement in the media and the surrounding clinics [36], recruiting walk-ins to the community pharmacy [37] or through pharmacist patient history review [38]. Two studies did not clearly report the recruitment process [39, 40].

All RCTs described the baseline assessment of all patients with regular follow-up of the intervention arm, with both baseline and follow up conducted by the pharmacist [38–40, 44]. The pharmacist attended the clinic appointment along with the physician in one study [38] and documented and communicated a plan to the physician in three studies [39, 40, 44]. In two studies, pharmacists also provided recommendations relating to laboratory testing and the need to prescribe new medications for undiagnosed conditions [38–40, 44, 45]. Lifestyle modification recommendations were provided by the pharmacist in five studies [38, 39, 41, 42, 44, 45], with only one of these measuring related outcomes using a nonvalidated questionnaire at three to six months [36] (Table 3).

#### Review objective 2: Impact of pharmacist role in MetS

Eight studies aimed to determine the impact of the pharmacist input. In the studies focusing on screening, the percentage of the newly diagnosed participants with MetS was reported, these participants would not have been diagnosed if the pharmacy screening service not been available [37, 41, 42]. These patients were followed-up by referral [41], or by communicating the relevant clinical parameters to the physician [36, 42]. One study demonstrated an improvement in the quality and quantity of documentation completed by physicians relating to MetS screening [43]. No further patient follows up was reported in one study [37].

The impact in MetS management in collaboration with the multidisciplinary team (MDT) was measured in four RCTs and one cross-sectional study, with improvement of anthropometric and metabolic parameters being the primary outcome measures [38–40, 44]. Additionally, determination of patient medication adherence was conducted in one study [44]. All but one study reported positive impact in terms of; achieving MetS parameter goals, reverting to non-MetS status, improved medication adherence and self-reported improved lifestyle modification. The study conducted in a psychiatric outpatient clinic failed to show significant improvement in metabolic parameters after the 12 months study period [40].

#### Review objective 3: The beneficiary population

Adults with comorbidities of MetS elements such as DM, HTN, dyslipidemia and obesity, were included in five studies [37–39, 44, 45]. Psychiatric patients receiving antipsychotic medications were targeted in three studies [40, 41, 45], since these medications are associated with significant weight gain [46]. One study included children and adolescents at high-risk of MetS, with a first-degree family history of type 2 DM, obesity or acanthosis nigricans [42], due to the potential link to underlying insulin resistance [47, 48]. Healthy volunteers were the subjects of one study [36] (Table 3).

#### Review objective 4: Facilitators and barriers

None of the studies specifically aimed to determine the facilitators and barriers to pharmacist input. Consequently, data relating to facilitators and barriers were extracted by the reviewers. Throughout all studies, the most commonly identified facilitator was effective communication, documentation and appropriate setting for MDT referrals, in addition to active collaboration with the MDT where each member of the MDT had a defined role (Table 4). Lack of funding for reimbursement of pharmacist time, purchasing consumables and other resources such as IT software was the most common barrier identified to the effective implementation of the pharmacist-led activity. Challenging behaviour of psychiatric patients was reported as a barrier in all studies involving psychiatric patients (Table 4).

**Table 4:** The facilitators and barriers of effective implementation of pharmacist input in MetS

	Schneiderhan et al., 2009 [41]	Olenak and Calpin, 2010 [36]	Benavides et al., 2010 [42]	Hammad et al., 2011 [38]	Plaster et al., 2012 [39]	kjeldsen et al., 2013 [43]	Via-Sosa et al., 2014 [37]	Schneiderhanet al, 2014 [40]	Azevedo et al. 2017 [44]	Ganzer, Nicole 2015 [45]	Total
*Facilitators*											
Collaboration with MDT with a defined role for each member	✓				✓	✓		✓	✓		5
Effective communication/documentation and referral to the MDT	✓	✓	✓	✓	✓	✓			✓	✓	8
Appropriate setting/easy accessibility		✓		✓			✓		✓	✓	5
Patient engagement in the therapeutic plan		✓		✓							2
Positive experience with pharmacist–led activity			✓								1
Pharmaceutical framework adaptation					✓				✓		2
Effective follow-up		✓		✓					✓	✓	4
Effective funds including; POC, advertising	✓	✓			✓		✓	✓			5
Authority to prescribe medications and order lab parameters										✓	1
*Barriers*											
Difficult behaviour of psychiatric patients	✓					✓		✓		✓	4
Lack of funding for consumables, staff and IT software		✓	✓	✓	✓	✓	✓	✓			7
Rural area and lack of healthcare access			✓								1
No consensus on the clear MetS definition for paediatrics			✓								1
Lack of formal integration of protocols in the healthcare system				✓							1
Restricting the pharmacist to dispensing					✓						1
Improper documentation and ineffective communication with physicians						✓					1

## Discussion

This is the first published systematic review focusing specifically on pharmacist input in MetS. This review identified ten studies, four of which were RCTs. The most frequently reported inputs were in screening and in management, with prevention-related activities described in one study. The main population studied was adults with comorbidities putting them at higher risk of developing MetS. Beneficial impacts were described in terms of earlier diagnosis, potentially earlier intervention and improvement in the MetS parameters. Successful integration with the MDT, effective communication and accessibility of the community pharmacies were most likely facilitators towards the implementation with lack of funding the most likely barrier.

This review adhered to best practice in conducting and reporting a systematic review, as described in “Preferred Reporting Items for Systematic Reviews and Meta-Analyses” (PRISMA) [49] (Online Appendix A). The wide range of patient populations reported in the studies may enhance the generalisability of findings to at-risk populations. The main review limitation was restricting the review to papers published in English, resulting in four studies not being included. While the quality of the studies was generally good, reporting could be enhanced by encouraging the authors to adopt robust reporting criteria such as those recommended by the EQUATOR (Enhancing the QUAlity and Transparency Of health Research) network [50, 51].

This systematic review has identified limited evidence upon which to inform the best practice of pharmacist input to MetS. The evidence base is derived from ten studies, only four of which were RCTs. Of the ten studies, there was marked variation in the aims and the models of care delivered, which significantly limits any potential for data pooling. Indeed, only four studies provided a comprehensive description of pharmacist interventions in terms of defined activities, training, processes, documentation, outcomes to be recorded and follow-ups.

A pharmacist-based intervention around MetS could be argued to be a complex intervention as defined by the UK Medical Research Council (MRC) which defines a ‘complex intervention’ as one with several interacting components, involving different behaviours and variability in outcomes [52]. The MRC complex intervention framework has four stages of development, feasibility/pilot testing, evaluation and implementation. It is worth noting that none of the ten studies in this systematic review included all these stages, with particular deficiencies around the development, feasibility and pilot testing stages. Ideally, the interventions should be developed and informed by evidence base in the literature (e.g. a systematic review), consider the theoretical basis for the intervention (e.g. behaviour change theory) and involve all stakeholders in development. Interventions developed according to this system are more likely to be successful compared to those developed pragmatically [52, 53]. There is also a lack of consideration of the MRC framework in the primary studies included in previous systematic reviews describing pharmacists input to managing Mets elements such DM [54], HTN [19], obesity [21] and cardiovascular risk factors [55].

Despite the absence of application of the MRC framework, this review has provided some evidence of the benefit of the pharmacist input, particularly in the screening for and management of MetS. There were positive outcomes of earlier diagnosis, referrals to the pertinent physician and reaching the MetS parameter goals.

Obese adults with chronic comorbid conditions and paediatrics with risk factors were identified in this review to be among the beneficiary populations. These findings concur with at-risk populations highlighted by international organizations. Moreover, the American Heart Association (AHA) and NHLBI underpin obesity and prediabetes as the main risk factors to develop MetS, in addition to other risk factors such as a sedentary lifestyle, atherogenic diet and older age [13]. This was further supported by the IDF communication consensus worldwide de-finition of MetS in 2006. Central obesity and insulin resistance were defined as the most potent risk factors to develop MetS, in addition to other risk factors such as; ageing, genetic predisposition, sedentary lifestyle, proinflammatory status and reproductive hormonal alteration [3]. Hence, prioritizing the at-risk population is logical and would be recommended, especially at the initial phase of implementing pharmacist-led activity with limited resource and experience.

Additionally, patients receiving antipsychotic medications were recognized by the American Psychiatric Association (APA) as at-risk population for development of MetS due to the strong association with weight gain, dyslipidemia and hyperglycemia, and emphasized the importance of regular screening and monitoring of MetS [56]. This supports the fact that psychiatric patients were also among the beneficiary populations identified in this review.

The challenges facing the implementation of the pharmacist within the collaborative service involving different specialities, including mental health, are common. While the nature of the conditions and the interventions are varied, the need for effective collaboration remains. The findings of a systematic review of 18 studies reporting the facilitators and barriers to the implementation of collaborative practice in mental health were in line with the findings of the current review. To successfully implement a new collaborative service, Wood and colleagues emphasised the importance of adopting a multidisciplinary approach in mental health, including a pharmacist, maintaining effective communications, applying structured care plans and sustaining active patient’s follow-up. On the other hand, the readiness of the organisations and staff for implementation and lack of knowledgeable, self-confident staff, adequate supervision and resources were the more pronounced barriers reported by Wood et al. [57].

A meta-synthesis of 29 qualitative studies categorized the influencing factors (facilitators and barriers) of implementing an advanced pharmacist run patient centred service into four categories; the patients’ factors, the interpersonal communication factors, organizational and community factors [58]. Among the most prominent factors enhancing implementation of advanced pharmaceutical services were easy accessibility of the service, sufficient resources for IT programmes, educational materials, service promotion, staff incentives, effective collaboration and communication and a predesigned protocol to define the role of each member of the team. The lack of these factors was barriers to implementation of the services [58]. This is similar to findings in this reported systematic review, for example in our study, organizational factors such as limited resources were also a barrier to implementation of pharmacist input to MetS; the interpersonal communication factors such as effective collaboration and communication with other healthcare providers were considered a facilitator, and specific patient factors including the challenging behaviour of psychiatric patients was a barrier to the practical implementation (Table 4).

The findings of this review are consistent with several published systematic reviews that have suggested that the MDT-pharmacist collaboration is the best model of care and facilitated the pharmacist’s role in screening and management of patients with MetS. Showande et al. confirmed the effectiveness of collaborative pharmacist management of Type 1 and Type 2 DM with 41 RCTs included in a systematic review and meta-analysis [54]. Similarly, in an earlier published systematic review by Altowaijri et al. across different settings (inpatient, outpatients and community pharmacies), pharmacist involvement with the MDT in secondary prevention of cardiovascular diseases was associated with better control of the cardiovascular risk factors and improvement in the clinical outcome [55].

Of paramount importance, emerging studies have suggested strategies to overcome the barriers to the implementation of collaborative pharmacist service. A meta-synthesis of 29 qualitative studies as well as the collaborative practice agreement issued by the national center for chronic disease prevention and health promotion, both have advocated utilizing evidence-informed practice along with seeking support from a leading champion in the field were suggested to alleviate the organisational and staff reluctance toward the implementation of new collaborative services. A multidisciplinary approach with engaging patients and their families was recommended to increase the readiness of the staff and patients to accept the pharmacist service. Emphasizing the potential long-term healthcare cost reduction secondary to the pharmacist collaboration and having more than one source of funding and cutting unnecessary expenses were suggested to overcome the financial barrier [59, 60].

### Implications for the further research phase

This systematic review highlighted the gap in the literature and provided evidence about the more effective model-of-care for the pharmacist to intervene in MetS. Future research is warranted to define the potential patient-centred model of care that should be systematically developed, evaluated, implemented and refined based on the MRC evaluation framework. Additionally, further qualitative research to explore in-depth the patients’ behaviours and health care professionals’ perception of the MDT collaborative practice will inform the development of a successful model of care.

## Conclusion

The limited number of studies describing pharmacist input in MetS provides some evidence of positive outcomes from screening and management as part of collaborative practice. Further work is required to provide more robust evidence of effectiveness and cost-effectiveness, while considering key barriers, to enable integration within standard practice.

## Electronic supplementary material

Below is the link to the electronic supplementary material.

Supplementary material 1 (DOCX 33 kb)

## Data Availability

Data sharing not applicable to this article as no datasets were generated or analysed during the current study.
